# Histopathologic and Radiographic evaluation of the electroacupuncture effects on ulna fracture healing in dogs

**Published:** 2014-04-25

**Authors:** H. Naddaf, A. Baniadam, S. Esmaeilzadeh, A.R. Ghadiri, M. Pourmehdi, H. Falah, O. Hosseini, F. Farmani, S. Sabiza

**Affiliations:** 1 *Department of Clinical Sciences, Faculty of Veterinary Medicine, Shahid Chamran University of Ahvaz, Ahvaz, Iran*; 2 *Department of Basic Sciences, Faculty of Veterinary Medicine, Shahid Chamran University of Ahvaz, Ahvaz, Iran*; 3 *Acupuncturist, Faculty of Medicine, Jondi Shapour University, Ahvaz, Iran*; 4 *Graduated of Veterinary Medicine, Faculty of Veterinary Medicine, Shahid Chamran University of Ahvaz, Ahvaz, Iran*

**Keywords:** Acupuncture, Dog, Histopathology, Radiography, Ulna

## Abstract

Acupuncture can affect bone healing by stimulation of sensory nerves and releasing of local and systemic neuropeptides. The purpose of this experimental study was to evaluate the effects of electroacupuncture on ulna fracture healing in dogs. In this study, 12 healthy dogs were randomly divided in to four equal groups, where group 1 was kept as control group and evaluated for 45 days, group 2: treatment group and evaluated for 45 days, group3: control group of 90 days and group 4: treatment group of 90 days. After induction of anesthesia, the ulna was cut with Gigli wire saw in each groups, 10 days after operation, the treatment (acupuncture) group was treated with 10 minutes electroacupuncture stimulations on the acupoints Kid1, Kid3, Kid6 and Kid7, for 10 days. Histopathologic samples of all dogs were harvested from bone osteotomized site in 45 and 90 days after surgery. Indices like, count of inflammatory cells, cartilaginous tissue, fibrotic tissue and deposition of collagen were evaluated on samples and classified with 0, 1, 2, and 3 degrees. Also, radiographic evaluation of the patients was applied using radiographic scoring system on days: 7, 15, 30, 45, 60 and 90 after surgery. This study revealed that, acupuncture had no effect on bone healing (p>0.05). Cause of non-significant difference changes between the control and treatment groups, and lack of complete healing in both groups may be due to lack of ulna bone fixation. Alternatively, selection of other acupoints in acupuncture could have a better healing role.

## Introduction

Acupuncture is among the many treatment options for pain in clinical veterinary practice. The advantages of acupuncture are, that it is practical, safe, less expensive, and with fewer side effects when compared with conventional pharmaceutical management of pain. The disadvantages are that a specific knowledge of the subject is necessary and that the response among individuals may vary (Cassu *et al.*, 2008).

The Philosophy of acupuncture is that there is energy called Qi. This energy is divided to two parts: Yang and Yin, which are balance in normal and healthy bodies, disturbance of this balanced condition by any reason causes disease. These energies are mobile in the body through 12 canals. There are some points on these canals, called accupoints.

The aim of acupuncture is recharging of the energy by stimulation of these points to produce a balanced condition (Schoen, 2001).

Electroacupuncture is a new method of acupuncture. In this method an electrical wave produced by electro acupuncture machine enters the body through the needle of acupuncture (Schoen, 2001). Electroacupuncture can even be of greater efficacy than dry needles alone as it produces a stronger, more prolonged, and uniform stimulation (Schofield, 2008).

Radius and ulna fractures are very common in small animals. It can be included in 5.8 to18% of fractures. Car accidents and minor trauma such as jumping or falling especially in small breeds are of most often causes of it (Muir, 1997; Hayashi et al., 2008). Especially, common problems of fractures of the ulna and radius in toy breeds are: non-union, delayed union, angulation and rotation of the two pieces of healed broken bones.

This is due to a decrease in vascular density in distal intersection of diaphyseal - metaphyseal than large breeds and with a poor prognosis for healing fractures in small-breed dogs. The biomechanical factors such as fracture and anatomical position may be more prone to instability after fracture stabilization. Nerve damage may also be associated with the initial fracture and radial nerve involvement should also be considered in dogs with ulna and radius fractures (Hayashi et al., 2008). Shen et al. (1999) associated the effect of acupuncture with pituitary-thyroid axis on radial fracture healing in rabbits. They observed a significant (p<0.01) increase in T4 and TSH of serum, when compared to the control group. Also, in the fourth week, T3 content of serum significantly increased (p<0.05) in comparison to the control group. Large intestine acupoints (LI), spleen (SP), gallbladder (GB) and stomach (ST), were stimulated. Sharifi et al. (2003) evaluated acupuncture treatment in the experimental radial fractures in dogs with orthopedic immobilization using casts. 10 minute daily stimulation of acupoints LI11, LI4, ST36, and TH5 were applied during the 2-week study. After 90 days, suitable and stimulatory effect was observed on callus formation which was relatively created bone tissue with trabecular structure and minimal fibrocartilage in the studied animals. They stated that acupuncture is effective in bone healing and callus formation (Sharifi *et al.*, 2003).

In traditional Chinese medicine it is assumed that bone healing activity is related to the kidney, not only in bone mineral supply, but also has great effects on bone growth of young animals. Ancient Chinese texts state that kidneys are responsible for bone health and survival (Xie and Preast, 2007). In this study we tried to promote and accelerate bone healing with the acupuncture induced stimulation of kidney related acupoints in dog.

## Materials and Methods

The project was approved by the local Committee of the Institutional Animal Care and use of Shahid Chamran University of Ahvaz. Twelve clinically healthy dogs of native breeds with the age of 16±4 months and average weight of 17±4.5 kg were used. Two weeks before the operation, anti-rabies (Merial Co, Czech republic) and DHPPi+L (BioVeta Co, Czech republic) vaccination was done, and also anti-parasitic treatment was done with the routine dose of Ivermectin 5% (Erfan Co, Iran), Mebendazole (Modava Co, Iran) and Peraziquantel (Damloran Co, Iran) and were separately kept for adaptations in the Veterinary hospital of Shahid Chamran University of Ahvaz.

All dogs were equally and randomly divided into four groups (each group= 3 dogs) where Group1 was kept as control group and evaluated for 45 days, Group 2: treatment group and evaluated for 45 days, Group 3: control group of 90days and Group 4: treatment group of 90 days.

All dogs were initially sedated using combination of acepromazine 1% (0.05 mg/kg, IM, Alfasan Co, Netherlands) plus ketamine (5 mg/kg, IM,). 15 minutes later, combination of ketamine 10% (10 mg/kg, IV, Alfasan Co, Netherlands) plus xylazine 2% (1 mg/kg, IV, Alfasan Co, Netherlands) (Tranquilli *et al.*, 2007) were used to induce anesthesia. Anesthesia was maintained with ketamine 10% (5 mg/kg, IV, Alfasan Co, Netherlands).

Surgical incision was applied with a length of 7 cm on the skin, just on the caudolateral (Brinker et al., 2006) aspect of the right ulna. Flexor carpi ulnaris and deep digital flexor muscles were dissected and the ulna was exposed. Then, a defect of 3mm in width was created on the one third of distal ulna using Gigli saw. Then, the muscles and skin of the surgical region were sutured.

Antibiotic therapy of all animals was done using cefazoline (22 mg/kg, IM) (Plumb, 2008). Ten days after surgery, all dogs mentioned in group 2 and 4 were electrically stimulated by electroacupuncture machine (XWD-808, Suzhou Huanqiu Acupuncture Medical Appliance, Beijing, China) on the acupoints *Kid1* (Hou-qiu or Yong-quan: On the volar side of the pelvic limb between the third and fourth metatarsals underneath the central pad), *Kid3* (Tai-xi: on the caudomedial aspect of the pelvic limb in the thin fleshy tissue between the medial malleolus of the tibia and calcaneus level with the tip of the medial malleolus”opposite and slightly distal to BL-60”), *Kid6* (Zhao-hai: on the caudomedial aspect of the pelvic limb in the depression immediately distal and caudal to the medial malleolus with the foot in dorsi flexion) and *Kid7* (Fu-liu: on the caudomedialaspect of the pelvic limb, 2 cun proximal to the kid 3, on the cranial border of the Achilles tendon) (Xie and Preast, 2007).

Administration of tramadol (5 mg/kg, PO) (Tranquilli *et al.*, 2007) was used in group 1 and 3. As an alternative analgesic drug and base of behavioral comfort, tramadol (5 mg/kg, PO), were assumed to be administered, if the animal of treatment group seemed to experience pain. In order to limit activity, all animals were caged inividually during the study. Electrical stimulation of 50 Hz, 0.3 mA, 1 ms with the needle of 30*0.25 mm (Suzhou Huanqiu Acupuncture Medical Appliance, Beijing, China) was used, 10 minutes daily and for 10 days (Feng et al., 2008). In order to assess the positive and negative effects of acupuncture on bone healing and callus formation, pathologic and radiographic evaluations were done.

### Pathologic evaluation method

At the end of study periods and under general anesthesia, Histopathologic samples of all dogs were harvested from bone osteotomized site in days 45 and 90 base of the defined groups with the average length of 2 cm (with a margins of 1 cm from the sides of the bone defect), and pain management was applied using tramadol (5 mg/kg, PO).

Then, right limbs of the sampled dogs were fixed using Robert-Jones bandage and caged for a little by one month. After evident ability to walk and general health, they were returned to nature again. Samples sustained within the required volume of 10% buffered formalin, and samples decalcification was done using chelating method by EDTA (Alers *et al.*, 1999).

Quantitative and qualitative criteria were used in pathologic evaluation. Scoring of the severity of the inflammatory reactions was performed according to inflammatory cells in the connective tissue. Thus, non-infiltration of inflammatory cells was equivalent to 0, diffuse infiltration of inflammatory cells was equivalent to1, the infiltration of inflammatory cells at the level of tissue detail visibility was equivalent to 2, and intense infiltration of inflammatory cells at the level of histological details invisibility was equivalent to 3.

Also, the grading of the cartilage and fibrous tissue formation was calculated on the basis of its size in comparison to the overall size of callus. In this way, total area of the callus and components of cartilage or fibrous tissue calculated using graticule grid homes and based on the number of houses occupied. Then, the percentage of each tissue was calculated. Ten percent of houses occupied of the total area of callus tissue was considered to +1, between 10-25% was graded as +2 and finally, between 25-40% and most of that was assumed +3 (Sharifi *et al.*, 2003).

### Radiologic evaluation

In order to evaluate healing trend in animals mentioned in groups 2 and 4, standard view radiographies of ulna were taken on days; 0, 7, 15, 30, 45, 60 and 90 after surgery.

It should be noted that, animals mentioned in group 1 and 2 were assessed up to 45 days while animals mentioned in group 3 and 4 were assessed up to 90 days.

In this way, after sedation of animals, caraniocaudal and mediolatteral view radiographies were taken (40 kvp, 30 mAs). At the end, radiographic assessment of fracture healing was done through radiographic scoring system as shown in Table 1 (Hayashi *et al.*, 2008). The experienced observer who evaluates the radiographic scores was blinded to the groups.

**Table 1:** Radiographic scoring system for assessment of fracture healing.

Scores	Radiograph signs
0	Presence of recent fracture with no bone formation.
1	Irregularity at fragment lines of fracture site.
2	Initial/discrete periosteal proliferation.
3	Exuberant/organized periosteal proliferation.
4	Exuberant osseous callus in evolution with presence of periosteal proliferation.
5	Exuberant osseous callus in evolution and discrete radiolucent line at gap between the fracture fragments.
6	Exuberant osseous callus and absent of radiolucent line.

At the end and after data collection, descriptive data analysis was performed using SPSS version19. To evaluate the effect of acupuncture on bone healing, repeated measures of ANOVA was used. Also, radiographic data was analysis using Kruskal-Wallis. The minimum level of significant was determined as p<0.05.

## Results

### Pathologic Evaluation

### Results for Wound Healing

In both control and Treatment groups, surgical wound of hand was healed 14 days after surgery without any complications and sutures were removed 14 days after surgery.

### Histopathology results

### Qualitative assessment results

### Control and treatment (acupuncture) groups, 45 days

**Fig. 1:**
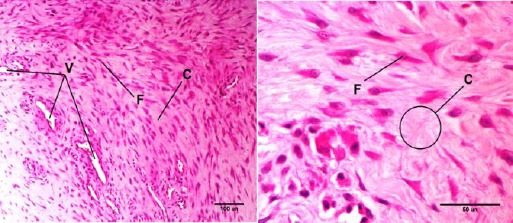
H&E staining of granulation tissue in bone defect region composed of fibroblast (F), collagen fibers (C), blood vessels (V). Right is related to control group, while left is related to treatment (acupuncture) group of 45days.

Microscopic examinations of control group showed the formation of new granulation tissue composed of many fibroblasts and a small amount of collagen (Figure 1) at the site of bone defect in all animals. This tissue was converted to the mature granulation tissue with most of the collagen fibers (Figure 1). In two samples, this tissue contains some mononuclear inflammatory cells.

**Fig. 2:**
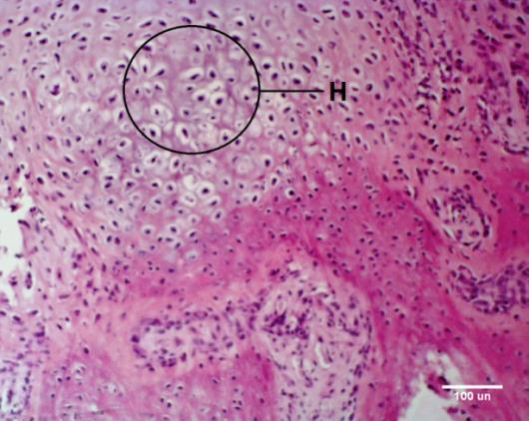
H&E staining of callus composed of cartilaginous centers (H).

Moreover, the cartilaginous centers were seen (Figure 2). Limited and small islands of bone within the membrane and the cartilage have also attracted attention.

Also in treatment group (acupuncture), adult granulation tissue in bone defect, is composed of blood vessels, fibroblast cell, a relatively large amount of collagen and a considerable amount of and mononuclear inflammatory cells (Figure 1). In two cases, a small number of cartilaginous centers were observed in defect while in the others, there were higher numbers. In one sample cartilaginous center was observed within cortical bone. Also in this samples would draw attention with the absence of inflammatory cells.

### Control and treatment (acupuncture) groups, 90days

**Fig. 3:**
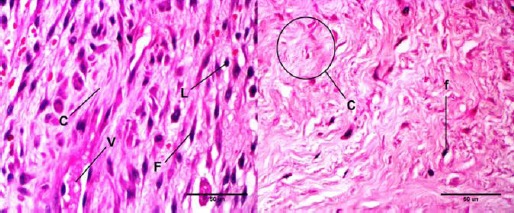
H&E staining of granulation tissue in bone defect region composed of fibroblast (F), collagen fibers (C), blood vessels (V) and lymphocyte (L). Right is related to control group, while left is related totreatment (acupuncture) group of 90 days.

Microscopic evaluation of the samples of control group showed, callus formation of the defect, composed of numerous collagen fibers and a small amount of fibroblasts, without the presence of inflammatory cells (Figure 3).

**Fig. 4:**
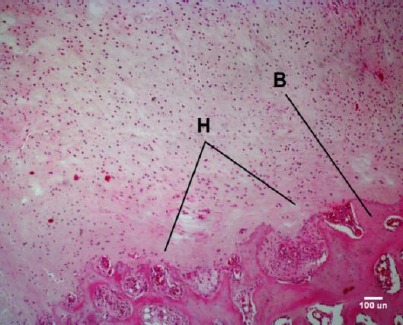
Formation of hyalan cartilage (H) in bone defect of control group 90 days. (B) is the edge of the broken bone.

In one sample, a major focus of cartilage was observed, which had two edges of the fracture together. In other cases, observed small focal cartilage in the defect edges, (Figure 4) and some of which were ossifying within the cartilage.

Also in treatment (acupuncture) group, mature connective callus and a considerable number of cartilage foci are seen. In two cases, a small center of ossifying in the cartilage and in others, major ossifying center was observed.

### Quantitative evaluation results

**Fig. 5:**
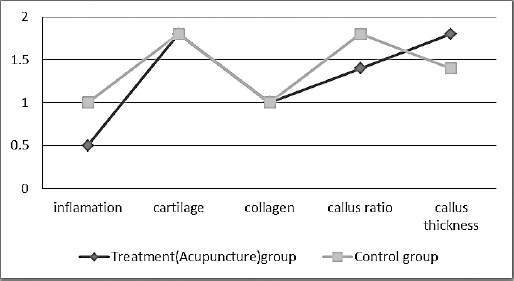
Histopathological parameters scoring of bone healing between the control and treatment (acupuncture) groups on day 45.

**Fig. 6:**
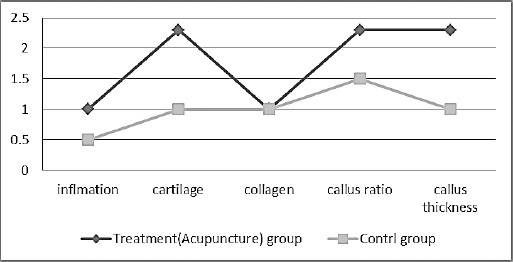
Histopathological parameters scoring of bone healing between the control and treatment (acupuncture) groups on day 90.

Quantitative evaluation results of mean and standard error of pathological index scoring of both control and treatment (acupuncture) groups are shown in Figures 5 and 6 and Table 2.

**Table 2:** Mean±SEM of pathologic index scoring in both control and treatment (acupuncture) groups

Pathologic Index	45 days		90 days	
	T	C	T	C
Inflammation	1±0	0.67±0.33	0.5±0.5	1±0
Cartilage	1.67±0.33	1.67±0.33	1±0	2.3±0.33
Collagen	1±0	1±0	1±0	1±0

Results revealed the effectiveness of the time on bone healing in both groups and as the time passed, the broken bone was healing (p<0.05). The Mann-Whitney test showed that the difference between periods of 45 and 90 days of two groups was not significant (p>0.05).

### 2-Radiographic Evaluation

Despite of some differences between Radiographic scoring, and increasing trend of bone healing in treatment (acupuncture) than in control group, all of them were not significant (p>0.05). Mean ± SD of Radiographic scoring system is shown in Table 3.

**Table 3:** Mean±SEM of radiographic scoring system for assessment of fracture healing in both control and treatment (acupuncture) groups

	Days	7	15	30	45	60	90
C	M	0.17	1.00	2.33	2.83	1.50	1.00
	SE	0.17	0.25	0.33	0.40	0.50	1.00
T	M	0.17	1.17	2.00	1.83	2.33	2.66
	SE	0.17	0.17	0.37	0.55	1.20	1.34

## Discussion

In the present study, Acupuncture did not lead to an increase in bone healing when compared to the control group, when evaluating the radiographic scores.

It seems that acupuncture could be related to be positive in initial vascular regrowth that is seen radiographically as demineralization.

The radiographic scores might not be sensible enough to detect any beneficial effect. This fact must be evaluated with another method such as bone biopsy, usually at experimental studies. Also the neuropeptides involvement in these cases after acupuncture must be investigated with immunohistochemical studies or serum detection and bone metabolism markers (Hayashi *et al.*, 2008).

Also, pathologic evaluation of the present study did not reveal any significant changes. Several conditions can influence bone healing after fracture, because it is realized by cells. These cells can be modified by almost all endogenous and exogenous factors that are related to cell metabolism.

Bone healing can be promoted by some factors like growth hormone, thyroid hormones, calcitonin, insulin, vitamin A and D, anabolic steroids, condroitin sulfate, hyaluronidase, anticoagulants, electric currents, oxygen and physical exercise. Growth factors like bone morphogenetic protein and platelet-derived growth factor are also important in accelerating bone healing (Millis, 1999).

Study of literature showed that the stimulation of acupuncture points and balance in the Yin and Yan energy in kidney meridians and maintaining this balance and the use of Qi energy on fracture site can activate Bone growth factors in the bleeding to establish the ossifying procedure (Hayashi *et al.*, 2008).

Some studies evaluated the effect of low-intensity pulsed produced by ultrasound, on callus maturation of rabbit bone defect healing and stated that an increase in density of callus was observed and an improvement in cortical bone healing were observed during the study. Then, they concluded that ultrasound pulses generated can shorten the time required for bone maturity (Hantes *et al.*, 2004; Tobita *et al.*, 2011).

The immunohistochemical demonstration of nerve fibers in the vicinity of bone tissue raises the possibility that neuropeptides may directly or indirectly modulate the activity of bone cells in physiological and pathological conditions, in line with the view of neuroendocrine and neuro immune interactions. Some nerve fibers where positive for SP, CGRP, VIP, NPY, tyrosine hydroxilase (TH) and interleukin-1 (IL-1) (Konttienen *et al.*, 1996; Lerner, 1996; Dawidson *et al.*, 1999).

Ma and Luo (2007) revealed that Electrical stimulation of acupuncture (EA) can increase vascular endothelial growth factor (VEGF) and consequently increase hormone-mediated angiogenesis and the formation of new vascular. Vascular endothelial growth factor in bone ossification within cartilages is more important when compared with the membranous ossification and also skeletal growth when compared with the bone healing. Also, they stated that the release and presence of this factor can be effective in healing of broken bones at the fracture site.

It is stated that, electrical acupuncture increases estradiol (E2), insulin-like growth factor1 (IGF-1) and insulin-like growth factor binding proteinBP1 (IGF-BPS). This increase causes calcium absorption and greater bone mineral density and thereby contributes to further improvement of the damaged area, which eventually bone fracture healing is accelerated. Exploration of Traditional Chinese Medicine (TCM) has shown that bone tissue is linked with kidney energy, which can stimulate bone healing (Feng *et al.*, 2008; Nakajima *et al.*, 2010). Growth and thyroid hormones are related to promote bone healing and can explain the good results with acupuncture and bone healing (Rogers *et al.*, 1992; Sharifi *et al.*, 2003).

Some neuropeptides like calcitonin gene-related peptide (CGRP), substance P (SP), vasoactive intestinal peptide (VIP), neuropeptide Y (NPY) are supposedly involved in bone growth, fracture repair and bone remodeling. The initial angiogenesis seems to be impossible without the influence and transmission of peptidergic fibers innervation. CGRP and VIP neuropeptides could be produced after acupuncture stimulation and released to a target organ (Hayashi et al., 2008), because they stimulate and degrade the activity of acupoints; B23 and K13 cause activity of kidney canal (Maciocia, 1996).

Hayashi et al. (2008) concluded that a group of animals that had received acupuncture analgesic, basically restored to better results in healing.

At the end, it should be noted that, probably cause of nonsignificant difference changes between the control group and test, and lack of complete healing in both groups is due to lack of ulna bone fixation.

Alternatively, selection of other acupoints in acupuncture could have a better healing role. The analgesic effects of acupuncture are better well understood than its ability to improved impaired function. Animals receiving analgesic treatment combined with acupuncture could lead to better results (Hayashi et al., 2008).

The results of the present study demonstrate that Acupuncture treatment did not accelerate bone healing in canine radius-ulna fracture during the 90 days of follow-up, as both radiographic and pathologic evaluations were not fully effective in this study.
